# Immune Responses in the Eye-Associated Lymphoid Tissues of Chickens after Ocular Inoculation with Vaccine and Virulent Strains of the Respiratory Infectious Laryngotracheitis Virus (ILTV)

**DOI:** 10.3390/v11070635

**Published:** 2019-07-10

**Authors:** Gabriela Beltrán, David J. Hurley, Robert M. Gogal, Shayan Sharif, Leah R. Read, Susan M. Williams, Carmen F. Jerry, Daniel A. Maekawa, Maricarmen García

**Affiliations:** 1 Poultry Diagnostic and Research Center, Department of Population Health, College of Veterinary Medicine, University of Georgia, 953 College Station Rd., Athens, GA 30602, USA; 2 Food Animal Health and Management Program, Department of Population Health, College of Veterinary Medicine, University of Georgia, 501 D. W. Brooks Dr., Athens, GA 30602, USA; 3 Veterinary Biosciences and Diagnostic Imaging, College of Veterinary Medicine, University of Georgia, 501 D. W. Brooks Dr., Athens, GA 30602, USA; 4 Department of Pathobiology, Ontario Veterinary College, University of Guelph, Guelph, Ontario, ON N1G 2W1, Canada; 5 Department of Veterinary Pathology, College of Veterinary Medicine, University of Georgia, 501 D. W. Brooks Dr., Athens, GA 30602, USA

**Keywords:** infectious laryngotracheitis virus (ILTV), conjunctiva-associated lymphoid tissue (CALT), Harderian gland (HG), viral genome load, interferon gamma, *Granzyme A*, interleukin-*12p40* gene, Interferon-*γ* gene

## Abstract

Infectious laryngotracheitis (ILT) is an acute respiratory disease of poultry caused by infectious laryngotracheitis virus (ILTV). Control of the disease with live attenuated vaccines administered via eye drop build upon immune responses generated by the eye-associated lymphoid tissues. The aim of this study was to assess cytokine and lymphocyte changes in the conjunctiva-associated lymphoid tissues (CALT) and Harderian gland (HG) stimulated by the ocular inoculation of the ILTV chicken embryo origin (CEO) vaccine strain and virulent strain 63140. This study offers strong evidence to support the roles that the CALT and HG play in the development of protective ILTV immune responses. It supports the premise that ILTV-mediated immunomodulation favors the B cell response over those of T cells. Further, it provides evidence that expansions of CD8α^+^ cells, with the concomitant expression of the *Granzyme A* gene, are key to reducing viral genomes in the CALT and halting ILTV cytolytic replication in the conjunctiva. Ultimately, this study revealed that the early upregulation of interleukin *(IL)-12p40* and Interferon (*IFN)-γ* cytokine genes, which shape the antigen-specific cell-mediated immune responses, retarded the decline of virus replication, and enhanced the development of lesions in the conjunctiva epithelium.

## 1. Introduction

Infectious laryngotracheitis (ILT) is a highly contagious acute respiratory disease of chickens caused by the avian alphaherpesvirus, *Gallid alpha herpesvirus 1* (GaHV-1), commonly known as infectious laryngotracheitis virus (ILTV). The disease has a worldwide distribution and is frequently observed in densely populated poultry production areas. Intervention strategies to control the disease rely on the implementation of biosecurity measures and vaccination with live-attenuated and/or recombinant viral vector vaccines expressing ILTV proteins [1]. Among live-attenuated vaccines, there are two main types: the tissue culture origin (TCO) vaccine [2] and chicken embryo origin (CEO) vaccines [3]. Both, CEO and TCO vaccines can be administered through the ocular, oral or intranasal mucosal routes to elicit local and systemic immunity [4,5], are capable of protecting chickens against clinical signs and mortality, and suppress replication of the challenge virus [6]. However, it has been demonstrated that live attenuated vaccine strains, most commonly the CEO vaccine strains, can revert to virulence if allowed to circulate in naïve chickens and give rise to outbreak viruses, such as the virulent strain 63140 [1]. Although live attenuated vaccines are routinely used in the field, there is little definitive knowledge about the molecular and cellular immune response associated with ocular vaccine-induced protection.

Early seminal studies demonstrated a critical role for the cell-mediated arm of the immune system in ILTV protection. In particular, it appears that specific T cell subsets play a role in vaccine protection and resistance against the disease [7,8,9]. More recent studies suggest that the local inflammatory processes contribute to the pathology of the disease, and eventually modulate the responses of the adaptive immune system to facilitate viral replication [10]. Furthermore, ILTV infection appears to down-regulate type I interferon transcription and delays the early transcription of inflammatory genes to favor initial virus replication in the trachea [11,12].

Unlike the mammalian immune system, the avian immune system lacks a discrete lymphatic network. Lymph nodes in mammals are the principal sites for initial antigen presentation in mounting naive responses. In contrast, chickens have a distributed immune network featuring clusters of mucosal-associated lymphoid tissues (MALTs) that appear to fill the role of organizing naïve responses [13]. The head-associated lymphoid tissue (HALT) is comprised of the nasal-associated lymphoid tissue (NALT), the eye-associated lymphoid tissues organized in the conjunctiva-associated lymphoid tissue (CALT) and the Harderian gland (HG). Despite the absence of a direct anatomic connection to the respiratory system, the HG, CALT, and NALT serve as major contributors of immunity for the upper respiratory airways. For example, in the NALT, cells capable of taking up Newcastle disease virus (NDV)-coated beads following intranasal administration were identified as potential antigen presenting cells (APCs) [14]. In the HG, the number of IgA- and IgY-producing cells increased after the ocular inoculation of an adenovirus 5 vector expressing the avian influenza H5 protein [15], and T effector cells (CD3^+^, CD44^+^) were detected in the HG and the CALT after infectious bronchitis virus (IBV) ocular inoculation [16].

Currently, little is known about the components of the cell-mediated immune responses associated with resistance against ILT that develops within the eye-associated lymphoid tissues. Recent findings indicate that the mucosal route of viral entry—ocular, oral, or nasal—can greatly influence the levels of ILTV lytic replication and lymphocyte infiltration in the trachea and conjunctiva [17]. Therefore, assessing the changes in immune cell populations within the eye-associated lymphoid tissues (CALT and HG) after ILTV exposure may help identify important features occurring in mucosal tissues that are involved in ILTV pathogenesis and host immune responses. To this end, the objective of the present study was to assess differences in immune cell dynamics by monitoring the number of T and B lymphocytes, the number of MHC class I and MHC class II antigen surface expressing cells, and transcript profile of interleukin *(IL)-12p40*, interferon *(IFN)-*γ, and *Granzyme A* genes in CALT and HG after ocular inoculation with the CEO vaccine strain or the virulent strain 63140. Collectively, this study highlights the important role of the eye-associated lymphoid tissue and the immunity elicited against ILTV infection. It provides evidence that the CALT and HG play different roles in the development of ILTV immune responses which were greatly influenced by the viral strain virulence. 

## 2. Materials and Methods

### 2.1. Ethical Statement

All animal experiments conducted in this study were performed under the Animal Use Proposal A2018 06-009-Y1-A0 approved by the Animal Care and Use Committee (IACUC) in accordance with regulations of the Office of the Vice President for Research at the University of Georgia.

### 2.2. ILTV Strains and Virus Titration

The 63140 virulent strain [18] and the CEO vaccine strain (Trachivax^®^ MERCK, Animal Health, Madison, NJ, USA) were utilized in this study. The virulent strain 63140 was propagated in chicken kidney cells obtained from 3 to 4-week-old specific pathogen-free (SPF) chickens [19]. The CEO vaccine strain was reconstituted as recommended by the manufacturers. Both viral strains were titrated in chicken kidney cells prepared from 3- to 4-wk-old SPF chickens. Kidney cells were disassociated in a 0.25% trypsin solution (Corning, Cellgro) at 37 °C. After trypsinization, cell pellets were resuspended in incomplete media (1x Ham’s media (Corning, Cellgro), 1× M 199 (Gibco), 2% tryptose phosphate broth, 0.62% 1M Hepes buffer, 1.2% Sodium bicarbonate (Sigma)). Chicken kidney (CK) cells density was adjusted to 8 × 10^5^ cells/mL in incomplete media with the addition of 10% fetal bovine serum (Atlanta Biologicals, Lawrenceville, GA, USA) and 2% antibiotic-antimycotic (Gibco). Fifty microliters of CK cells was seeded in 96-well plates. Twenty-four hours post-seeding, the complete medium was replaced with incomplete media, and plates were used 48 h after seeding. Five replicates of virus dilutions ranging from 10^−1^ to 10^−9^ were inoculated per well with 50 microliters of each virus dilution. Plates were incubated at 39 °C and 5% CO_2_ for 5 days. The 50% tissue culture infective dose (TCID_50_) was determined by the presence of virus-induced cytopathic effect and estimated by the Reed and Muench method [20].

### 2.3. Experimental Design and Time Line

A total of 215 specific pathogen-free (SPF) fertile White Leghorn eggs obtained from Charles River (Norwich, CT, USA) were incubated and hatched at the Poultry Diagnostic Research Center (PDRC, Athens, GA, USA), University of Georgia hatchery facilities. One-day-old chicks were housed in isolation units, with filtered air and negative pressure, and provided with feed and water ad libitum. At five weeks-of-age, chickens were arbitrarily divided in three groups. One group of chickens was inoculated with virulent strain 63140 (*n* = 75); a second group was inoculated with the CEO vaccine strain (*n* = 75). Both virus strains were administered via the ocular route. Viruses were diluted in incomplete media and a total dose of 10^3.5^ TCID_50_ was administered per chicken in a 60 μL volume, 30 μL per eye. The third group of chickens (*n* = 65) was mock inoculated with cell culture media in a similar fashion and used as negative control. At 1, 3, 5, 7, and 9 days post-inoculation (dpi), conjunctiva-associated lymphoid tissue (CALT) of the lower eyelids, Harderian gland (HG) from both eyes, and upper trachea sections were collected from five chickens from each of the three treatment groups. CALT and HG samples were used for flow cytometry analysis, while the upper trachea sections were used to determine viral genome load by real-time PCR. Additionally, CALT and HG samples were collected from 4 to 6 chickens per group at 1, 3, 5, 7, and 9 dpi and utilized to assess viral genome load and host cytokines genes transcription levels. At 1, 3, 5, and 7 dpi conjunctiva samples were collected from five chickens from each group for histopathology examination. Clinical signs were obtained from days 2 to 7 post-inoculation (pi) from 6 to 7 randomly selected chickens per group. Signs of respiratory distress, lethargy and conjunctivitis were scored as previously described [6]. Briefly, signs of dyspnea, conjunctivitis and lethargy were scored on a scale of 0 to 3—normal (0), mild (0.5 to 1), moderate (1.5 to 2), and severe (2.5 to 3)—for each of the 3 clinical categories (Total maximum score = 9). Each chicken received a total clinical sign score, which was the sum of individual clinical signs categories. A total mean clinical signs score was calculated per group at each time point post-infection.

### 2.4. Samples Collection 

Chickens were euthanized by CO_2_ gas inhalation for one minute. The CALT tissues from both lower eyelids and HG from each eye were aseptically removed and immediately placed in 15 mL conical tubes containing 8 mL of ice-cold transport medium (calcium and magnesium-free PBS with 0.5% bovine serum albumin) (Thermo Fisher Scientific, Waltham, MA, USA). Samples were kept on ice until the tissues were processed for flow cytometry analysis. The upper trachea was collected and immediately placed in 1 mL of sterile phosphate-buffered saline solution (HyClone, Logan, UT, USA) with 2% antibiotic-antimycotic 100X (Gibco, Grand Island, NY, USA), and 2% fetal bovine serum (FBS, Atlanta^®^, Biological, Flowery Branch, GA, USA) and stored at −80 °C until processing for DNA extraction. Additional CALT and HG samples were collected and placed in Lysing matrix D tubes (MP Biomedical, Santa Ana, CA, USA) containing 1 mL of TRIzol^®^ (Thermo Fisher, Waltham, MA, USA). These were incubated for 30 min on ice and homogenized using a FastPrep-24™5G (MP Biomedical, Santa Ana, CA, USA) and immediately stored at −80 °C until processing for RNA and DNA extraction.

### 2.5. Nucleic Acid Extractions and cDNA Synthesis

Extraction of RNA and DNA were performed from the CALT and HG TRIzol^®^ homogenates. Briefly, immediately after the homogenization of CALT and HG samples in 1 mL of TRIzol (Thermo Fisher, Waltham, MA, USA), 200 μL of chloroform was added and the samples were centrifuged at 4 °C for 15 min at 12,000× *g*. Total RNA was further purified from the upper aqueous phase using the RNeasy kit (QIAGEN, Valencia, CA, USA) as previously described [12]. To eliminate DNA contamination, the eluted RNA was treated with TURBO DNA-free™ (2 to 4 units/per reaction) (Ambion, Carlsbad, CA, USA) for 30 min at 37 °C. The DNase was inactivated with 5 μL of DNase inactivation buffer for 2 min at 23 °C. After DNA digestion, a total of 80 units of RNase inhibitor (RNase OUT™, Invitrogen, Carlsbad, CA, USA) was added per sample. To assess the purity and concentration of RNA per sample, optical density ratios (260/280 and 260/230) were obtained using the NanoDrop™ 2000c (Thermo Fisher Scientifics, Waltham, MA, USA). Previous to cDNA synthesis, a real time PCR reaction that amplifies the chicken β-actin gene was performed to ensure the absence of residual DNA in the eluted RNA using primers previously described [12]. The β-actin gene amplification reaction was performed in a 20 μL volume—10 μL of SYBR^®^ Select Master Mix (2X) (Life Technologies, Carlsbad, CA, USA), 1 μL of 5 μM of each primer, 5 μL of template and 3 μL of nuclease free water. The cycling profile was 50 °C for 2 min; 95 °C for 2 min; 40 cycles of 95 °C for 15 s; and 60 °C for 1 min. 

Complementary DNA (cDNA) synthesis was performed in 20-μL reactions including 250 to 500 nanograms of RNA, 200 units of reverse transcriptase (SuperScriptIII™ RT Invitrogen Life Technologies, Carlsbad, CA, USA) and 200 nM oligo (dT) 15 primers (Sigma-Aldrich, St. Louis, MO, USA). The reverse transcription was performed following the First-Strand Synthesis System protocol as recommended by manufacturer’s (Invitrogen Life Technologies, Carlsbad, CA, USA). The cDNA was stored at −20 °C until further real time PCR analysis.

Total DNA from CALT and HG was extracted from the organic phenol phase of the TRIzol^®^ homogenate following the manufacturer’s recommendations. Briefly, 300 μL of absolute ethanol was added to the phenol phase, followed by centrifugation at 2000× *g* for 5 min at 4 °C. The pellet was washed twice with 1 M sodium citrate in 10% ethanol and once with 75% ethanol and centrifuged at 2000× *g* for 5 min at 4 °C and re-suspended in 200 μL of 8 mM sodium hydroxide. DNA purification was performed using the MagaZorb^®^ DNA extraction mini-prep 96 well kit following the manufacturer´s recommendations (Promega, Madison, WI, USA) as previously described [21]. Eluted DNA was stored at −80 °C until further real time PCR analysis. 

### 2.6. Histopathological Examination of Conjunctiva Tissues 

At 1, 3, 5, and 7 days post-ocular inoculation, conjunctiva tissues were collected and placed in 10% buffered natural formalin and fixed for 24 h at room temperature. Routine tissue processing, embedding and sectioning was performed. Four-micrometer sections stained with haematoxylin and eosin (H&E) were subjected to microscopic examination for the signs of ILTV lytic replication, indicated by the presence of syncytial cell formation and eosinophilic intranuclear inclusion bodies.

### 2.7. Single-Cell Suspension Preparation

Single-cell suspensions from CALT and HG tissues were obtained by mechanical disruption using a 60-µm wire screen mesh (Sigma-Aldrich, St. Luis, MO, USA). CALT cell suspensions were passed once through a 70-µm cell strainer (Thermo Fisher Scientific, Waltham, MA, USA), washed once with transport medium, and centrifuged at 250× *g* for 7 min at 4 °C. The HG cell suspension was passed once through a 70-µm and twice through a 40-µm cell strainer and washed twice as described above. CALT cells were re-suspended in 1 mL and HG in 2 mL of transport medium, cells were enumerated, and viability was determined using trypan blue exclusion on a Cellometer Mini (Nexcelcom Bioscience, Lawrence, MA, USA). CALT and HG cells suspensions were adjusted to 1 × 10^6^/100 µL and 4 × 10^6^/100 µL, respectively.

### 2.8. Antibody Dilutions and Staining Procedures

Lymphocyte populations from each tissue were evaluated using four monoclonal antibody staining combinations: (1) Allophycocyanin (APC) conjugated to mouse anti-chicken CD45, a pan leukocyte marker alone (Clone LT40, Southern Biotech, Birmingham, AL); (2) Phyocoerythrin (PE) conjugated to mouse anti-chicken CD4 (Clone CT-4) paired with fluorescein isothiocyanate (FITC)-conjugated to mouse anti-chicken CD8α (Clone CT8) and APC conjugated mouse anti-chicken CD45; (3) PE-conjugated mouse anti-chicken IgM (Clone M-1) paired with FITC-conjugated mouse anti-chicken IgA (Clone A-1, Southern Biotech, Birmingham, AL, USA) and APC conjugated mouse anti-chicken CD45; (4) PE-conjugated mouse anti-chicken MHCII (Clone 2G11) paired with FITC-conjugated mouse anti-chicken MHCI (Clone F21-2, Southern Biotech, Birmingham, AL, USA) and APC conjugated mouse anti-chicken CD45. Working dilutions of antibodies were performed in FACS buffer containing calcium and magnesium-free PBS, 0.5% bovine serum albumin, with 0.1% sodium azide. Antibody concentrations were optimized to the minimum saturating and cross-talk (between colors) concentrations in prior trials. The APC-CD45 was used at a final concentration of 0.05 μg per reaction, PE-CD4 at 0.25 μg, FITC-CD8 α at 0.5 μg, PE-IgM at 0.5 μg, FITC-IgA 1 μg, PE-MHCII 0.125 μg, and FITC-MHCI at 0.5 μg. CALT and HG cell suspensions were distributed in round-bottom 96-well plates, 100 μL per well and incubated with 100 μL of antibody combinations and incubated for 30 min at 4 °C in a rotator plate shaker. After incubation, plates were centrifuged at 4 °C for 7 min at 250× *g*, cell pellets were washed once with 200 μL of FACS buffer and centrifuged at 4 °C for 5 min at 250× *g*. Cells were then re-suspended in 100 μL of FACS buffer and 100 μL of Intracellular (IC) fixation buffer (Thermo Fisher Scientific, Waltham, MA, USA) and stored overnight at 4 °C. Prior to flow cytometry analysis fixed cell suspensions were diluted with 200 µL of calcium and magnesium-free PBS.

### 2.9. Flow Cytometry Analysis

**Figure 1:**
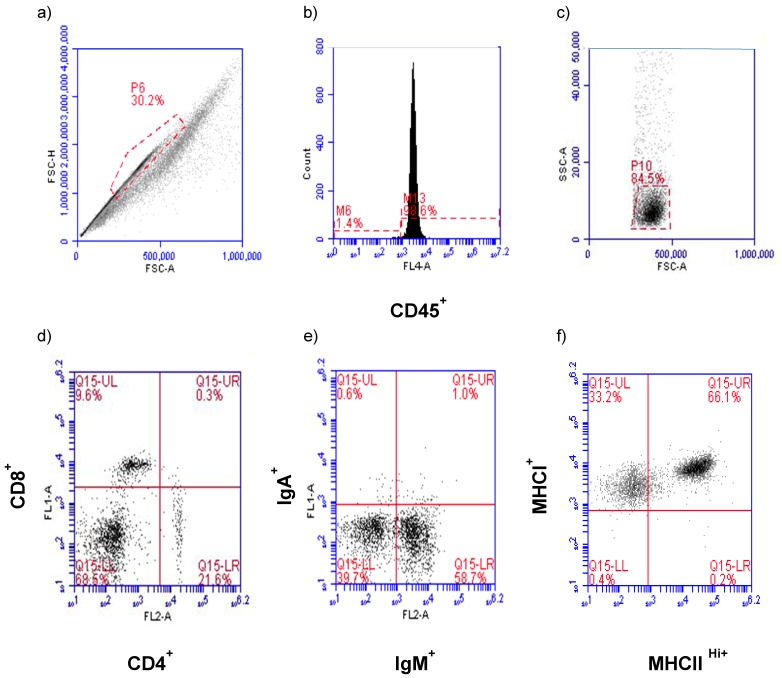
Gating strategy of the lymphocyte population. Primary gating was based on size consistency and single cell using forward scatter height versus forward scatter area (FSC-H vs. FSC-A) (**a**) and subjected to CD45 assessment (**b**). Representative flow cytometric forward-angle and right-angle scatter (FSC-A vs. SSC-A) gated on only CD45^+^ cells were used to identify the lymphocyte population (**c**). The CD45^+^ lymphocytes population was subjected to CD4^+^ and CD8α^+^ (**d**), IgM^+^ and IgA^+^ (**e**), MHCI^+^ and MHCII^Hi+^ (**f**) assessment.

Flow cytometry analysis and data collection were performed on a BD Accuri C6 Flow Cytometer and software (ver 1.0.264.21) (San Jose, CA, USA). Gating for leukocyte populations in HG and CALT and the establishment of control windows for single staining and double staining were determined beforehand. Briefly, primary gating was based on size consistency and single cells using forward scatter height versus forward scatter area (FSC-H vs. FSC-A) (Figure 1a). Cells were then subjected to CD45 assessment to establish the leukocyte population in this primary gate (Figure 1b). Forward and right-angle scatter (FSC-A vs. SSC-A) relative to CD45^+^ expression was used to identify the lymphocyte population and exclude macrophages from the analysis (Figure 1c). The CD45^+^ lymphocytes were then evaluated for CD4^+^, CD8α^+^ (Figure 1d), IgM^+^, IgA^+^ (Figure 1e), and MHC class I^+^, MHC class II^Hi+^ (Figure 1f) antigen expression. Using the lymphocyte electronic gate, 4000 and 3000 events were collected per CALT and HG, respectively. 

### 2.10. Quantification of Viral Genome Load 

The detection of ILTV genomes in samples was determined by a duplex probe-based-Real-time PCR [22]. The duplex assay amplifies the UL44 (gC) viral gene and a fragment of the chicken α2-collagen gene. The relative amount of viral DNA per samples was expressed as log_10_ 2^−ΔΔ*C*t^ value.

### 2.11. Transcription of Host Cytokine Genes

To assess how the cell-mediated responses to ILTV in the CALT and HG was formed, transcription of the chicken *IL-12p40*, *IFN-γ* and *Granzyme A* genes were quantified. Briefly, 5 µL of 1:10 dilutions of previously produced cDNA were assembled in 384-well plates (Roche Diagnostics, Indianapolis, IN, USA) in 20 μL reactions containing LightCycler 480 SYBR Green I Master mix (Roche Diagnostics GmbH) 0.25 µM of each gene-specific primer set, the chicken β-actin primer set. Real-time PCRs were performed in the LightCycler 480II instrument (Roche Diagnostics, Indianapolis, IN, USA). Primers, amplification reaction parameters and efficiencies for each reaction are shown in Table 1. The *IL-12p40*, *IFN-γ* and *Granzyme A* genes relative transcription changes were determined as compared to the mock-inoculated group of chickens, using the chicken β-actin transcript as an endogenous control, and presented as median fold change (log_10_ 2^−ΔΔ^*^C^*^t^) and 95% confidence interval [12].

### 2.12. Statistical Analysis

The non-parametric Mann–Withney U test (*p* ≤ 0.05) was utilized to evaluate differences between CEO and 63140 genomes load in conjunctiva, Harderian gland and trachea at each time point post-inoculation (pi). The mean and median of each set of assessments did not show differences, thus the results are presented as mean with standard deviation (SD). The one-way analysis of variance Kruskal–Wallis test and the Dunn´s multiple comparison post-test (*p* ≤ 0.05) were utilized to individually compare CD4^+^, CD8α^+^, IgM^+^, IgA^+^ and MHCI^+^/MHCII^Hi+^ percentage changes elicited after 63140, CEO, and mock inoculation in the CALT and HG. The mean and median for the cell surface markers did not show differences, thus the results are presented as the mean with standard deviation (SD). The non-parametric Mann–Withney U test (*p* ≤ 0.05) was utilized to assess differences in median fold changes for *IL-12p40*, *IFN-γ*, and *Granzyme A* genes transcription elicited by ocular inoculation with CEO and 63140 ILTV strains. Median fold change and 95% confidence intervals at each time point post-inoculation (pi) are presented. Pearson correlation coefficient (r) were calculated to determine the strength of the association between: MHCI^+^/MHCII^Hi+^ and IgM^+^ cell percentages, *IL-12p40* and *IFN-γ* genes transcription, and *Granzyme A* gene expression and the number of viral genomes. Statistical analysis of the data was conducted using the Prism 7 (GraphPad, Software Inc. San Diego, CA, USA).

## 3. Results

### 3.1. Clinical Signs

**Figure 2:**
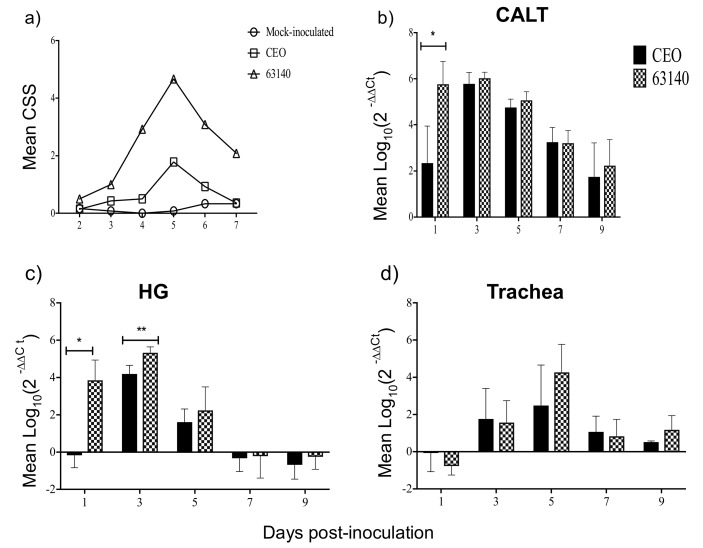
Mean clinical sign scores (CSSs) and mean genome load (Log_10_ 2^−ΔΔ*C*t^) of the chicken embryo origin (CEO) vaccine strain and 63140 virulent strain in the conjunctiva-associated lymphoid tissue (CALT), Harderian gland (HG) and trachea. (**a**) Mean clinical sign scores recorded from 2 to 7 dpi (*n* = 6 to 7). Mean clinical sign scores are indicated by geometrical symbols. Mean viral genome load in CALT (**b**), HG (**c**), and trachea (**d**) measured at 1, 3, 5, 7 and 9 dpi. The bars represent the mean viral genome load and the vertical lines indicate the standard deviation for each tissue. Significant differences in viral genome load were measured between strains on the basis of the tissue assessed at each time point as are indicated (* *p* ≤ 0.0332, ** *p* ≤ 0.0021).

Throughout the study, no chickens were euthanized due to the severity of clinical signs and no spontaneous deaths were documented post-ocular inoculation with either the CEO vaccine strain or the virulent 63140 strain. However, from 4 to 7 days post-inoculation (dpi), although mortalities were not recorded, the group of chickens inoculated with the 63140 virulent strain showed markedly higher clinical sign scores than chickens inoculated with the CEO vaccine strain (Figure 2a). The peak of clinical signs occurred at 5 dpi with mean total clinical signs score of 4.66 and 1.7 for the 63140 and CEO-inoculated groups, respectively (Figure 2a).

### 3.2. Viral Genome Load

Throughout the course of the infection the viral genome load of both the CEO vaccine strain and the 63140 virulent strain were markedly higher in the CALT (Figure 2b) than in the Harderian gland (Figure 2c) or the trachea (Figure 2d). In the CALT, at 1 dpi, the 63140 strain mean genome load was 5.76 log_10_, while the CEO vaccine strain mean genome load was 2.34 log_10_ (*p* ≤ 0.0332). Between 3 to 9 dpi, no differences in CEO and 63140 genomes load were detected. The CEO (5.78 log_10_) and 63140 (6.0 log_10_) mean genome load peaked in the CALT at 3 dpi, began declining at 5 dpi, and mean genome load of 1.75 log_10_ and 2.20 log_10_ were detected in CEO and 63140 inoculate groups, respectively, by 9 dpi (Figure 2b). In the HG, at 1 dpi, no evidence of CEO genome load was detected, but a mean 63140 genome load of 3.85 log_10_ was obtained. The CEO (5.3 log_10_) and 63140 (4.19 log_10_) mean genome load peaked in the HG at 3 dpi, began declining at 5 dpi, and genomes were eliminated by 7 dpi (Figure 2c). In the trachea, genomes were first detected at 3 dpi. On 5 dpi, the 63140 strain mean genome load was 4.20 log_10,_ and the mean genome load of the CEO vaccine strain was 2.48 log_10_. Both CEO and 63140 genomes persisted in the trachea on 7 and 9 dpi (Figure 2d). No viral genome was detected in any of the tissues collected from mock-inoculated group of chickens.

### 3.3. Histopathological Changes in the Conjunctiva Epithelium

**Figure 3:**
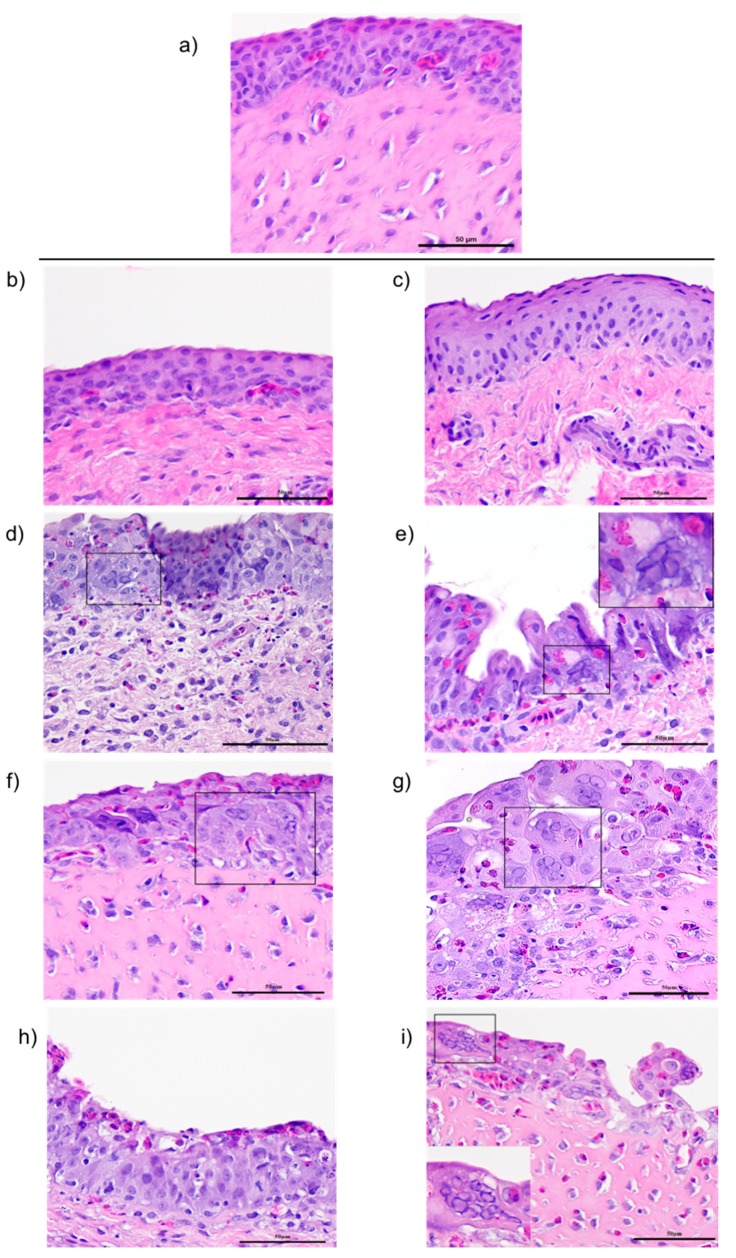
Photomicrographs of conjunctiva epithelium collected from the CEO vaccine strain- and virulent 63140 strain-inoculated chickens. Conjunctiva sections from the mock-inoculated group (**a**), from CEO vaccine strain-inoculated chickens collected at 1 (**b**), 3 (**d**), 5 (**f**), and 7 (**h**) dpi and from 63140-inoculated chickens collected at 1 (**c**), 3 (**e**), 5 (**g**), and 7 (**i**) days post-inoculation. Evidence of infectious laryngotracheitis virus (ILTV) lytic replication; syncytia cell formation with eosinophilic intranuclear inclusion bodies are indicated in insets within the photomicrograph.

Compared to the mock-inoculated group (Figure 3a), the histopathological examination of the conjunctiva epithelium of chickens inoculated with the CEO vaccine strain (Figure 3b) and 63140 virulent strain (Figure 3c) showed minimal tissue damage, absence of syncytial cell formation, and no eosinophilic intranuclear inclusion bodies. At 3 dpi, the conjunctiva epithelium from both CEO-(Figure 3d) and 63140-inoculated (Figure 3e) chickens showed focal syncytia cell formation with eosinophilic intranuclear inclusions, and mild to moderate heterophilic infiltration. At 5 dpi, numerous syncytial cells with eosinophilic intranuclear inclusion bodies were present in the conjunctiva epithelium of both CEO (Figure 3f) and 63140 (Figure 3g) inoculated birds. In addition, mild multifocal heterophilic infiltrates were detected in the conjunctiva epithelium of the CEO vaccine strain inoculated chickens (Figure 3f), while moderate infiltrates of lymphocytes and heterophils, cell degeneration, and necrosis and sloughing of epithelial cells were observed in the conjunctiva epithelium of the 63140 virulent strain inoculated chickens (Figure 3g). At 7 dpi, the conjunctiva epithelium of CEO-inoculated chickens showed some multifocal necrotic epithelial cells, moderate infiltrates of heterophils and lymphocytes, but an absence of syncytial cell formation or eosinophilic intranuclear inclusion bodies (Figure 3h). Whereas, the conjunctiva epithelium of the 63140-inoculated chickens still presented syncytial cells, eosinophilic intranuclear inclusion bodies, marked necrosis, sloughing of the mucosa epithelium and mild heterophilic infiltrate (Figure 3i).

### 3.4. Dynamics of IgM^+^, MHCI^+^/MHCII^Hi+^ and IgA^+^ Cells in CALT and HG of ILTV-Infected Chickens

**Figure 4:**
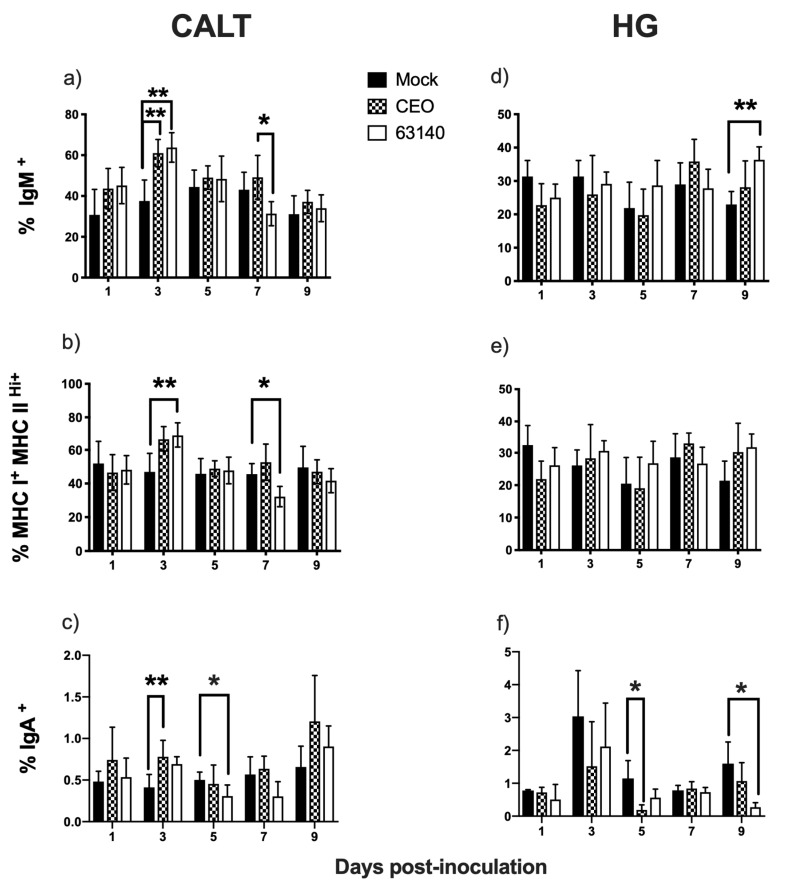
Percentages of IgM^+^, MHCI^+^/MHCII^Hi+^ and IgA^+^ cells in the conjunctiva-associated lymphoid tissue (CALT) and Harderian gland (HG) of chickens inoculated with the CEO vaccine strain and virulent 63140 strain. Percentage of IgM^+^ (**a**,**d**), MHCI^+^/MHCII^Hi+^ (**b**,**e**), and IgA^+^ (**c**,**f**) cells in CALT 1 to 9 days post-CEO (**a**–**c**) and 63140 (**d**–**f**) inoculation. The one-way analysis of variance Kruskal–Wallis test and the Dunn´s multiple comparison post-test (*p* ≤ 0.05) were performed to individually compare IgM^+^, IgA^+^ and MHCI^+^/MHCII^Hi+^ percentages in CALT and HG for mock-, CEO-, and 63140-inoculated groups. The mean percentage is indicated by bars and vertical lines represent the standard deviation (SD). Significant increase in IgM^+^, MHCI^+^/MHCII^Hi+^, and IgA^+^ cells is indicated by (**), and significant decrease by (*) (*p* ≤ 0.05).

The percentages of IgM^+^ B cells in the CALT of CEO- and 63140-inoculated birds at 3 dpi were significantly (*p* ≤ 0.05) higher than the IgM^+^ B cells percentage detected in the mock-inoculated birds (Figure 4a). Similarly, at 3 dpi a significant difference (~68% vs. 48%) (*p* ≤ 0.05) was observed for the percentage of MHCI^+^/MHCII^Hi+^ cells in the CALT of the 63140-inoculated birds and the mock-inoculated birds. While the CEO-inoculated birds showed a numerically higher percentage of IgM^+^ B cells than the mock-inoculated birds, but this was not significantly different (Figure 4b). At 7 dpi, the percentage of IgM^+^ and MHCI^+^/MHCII^Hi+^ cells significantly (*p* ≤ 0.05) decreased in the 63140 birds, but not for the CEO-inoculated birds (Figure 4a,b). Changes in IgM^+^ and MHCI^+^/MHCII^Hi+^ cells, from 1 to 9 dpi, for both the 63140 and CEO groups of birds were very strongly correlated with coefficients (r) of 0.998 and 0.957 (*p* < 0.0001), respectively. Concurrent with the observed patterns of IgM^+^ and MHCI^+^/MHCII^Hi+^ cells, the percentage of IgA^+^ cells in the CALT of the CEO-inoculated birds significantly (*p* ≤ 0.05) increased at 3 dpi. Although not significantly different from the mock-inoculated birds, the percentage of IgA^+^ cells increased again at 9 dpi in the CALT. For the 63140-inoculated birds, the IgA^+^ cells percentage in the CALT significantly (*p* ≤ 0.05) decreased at 5 dpi, and continued to decline through 7 dpi (Figure 4c). This coincided with the decline of IgM^+^ and MHCI^+^/MHCII^Hi+^ cells (Figure 4a,b).

In the HG, changes in IgM^+^ and MHCI^+^/MHCII^Hi+^ cells for 63140 and CEO-inoculated birds were smaller than those detected in the CALT. The IgM^+^ and MHCI^+^/MHCII^Hi+^ cells increased at 5 dpi for the 63140-inoculated group, and at 7 dpi for the CEO-inoculated group. These increases were not significantly different from the mock-inoculated birds. The percentage of IgM^+^ cells for the 63140-inoculated group significantly (*p* ≤ 0.05) increased at 9 dpi (Figure 4d) and, although not significant different from the mock-inoculated birds, the MHCI^+^/MHCII^Hi+^ cells also increased (Figure 4e). Similarly, in the CEO-inoculated birds, both IgM^+^ and MHCI^+^/MHCII^Hi+^ cells were numerically increased at 9 dpi (Figure 4d,e).

The IgA^+^ cells in the HG at 3 dpi demonstrated their highest levels for both the CEO- and 63140-inoculated groups, but they were not significantly different from the mock-inoculated birds. The IgA^+^ cells were significantly (*p* ≤ 0.05) decreased for the CEO birds at 5 dpi, and for the 63140 birds at 9 dpi (Figure 4f). No relevant association was found between IgM^+^ and IgA^+^, or MHCI^+^/MHCII^Hi+^ and IgA^+^ cells in the CALT or the HG for neither the 63140- nor CEO-inoculated birds.

### 3.5. Dynamics of CD4^+^ and CD8α^+^ Cells in the CALT and HG of ILTV-Infected Chickens

**Figure 5:**
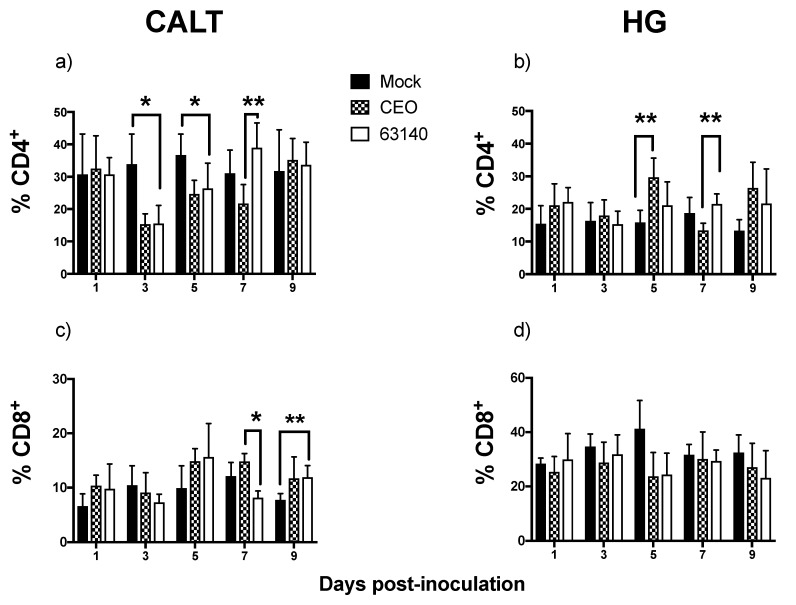
Percentages of CD4^+^ and *CD8*α*^+^* cells in the conjunctiva-associated lymphoid tissue (CALT) and Harderian gland (HG) of chickens inoculated with the CEO vaccine strain and 63140 virulent strain. Percentage of CD4^+^ in CALT (**a**) and in HG (**b**); percentage of CD8^+^ in CALT (**c**) and HG (**d**) from 1 to 9 days post-CEO and 63140 inoculation. The one-way analysis of variance, Kruskal–Wallis test and the Dunn´s multiple comparison post-test (*p* ≤ 0.05) were performed to individually compare CD4^+^ and *CD8*α*^+^* cell percentages in CALT and HG for the mock-, CEO,- and 63140-inoculated groups. The mean percentage is indicated by bars and vertical lines represent the standard deviation (SD). Significant increase in CD4^+^ and *CD8*α*^+^* cells is indicated by (**), and significant decrease by (*) (*p* ≤ 0.05).

The CD4^+^ cells in the CALT of 63140-inoculated birds significantly (*p* ≤ 0.05) decreased at 3 and 5 dpi. This was followed by a significant increase (*p* ≤ 0.05) at 7 dpi (Figure 5a). For the CEO-inoculated birds, the CD4^+^ cells also declined at 3 dpi, but this was not significantly different from the mock-inoculated birds. In the HG, the CD4^+^ cells significantly (*p* ≤ 0.05) increased for the CEO and 63140-inoculated birds at 5 and 7 dpi, respectively (Figure 5b).

The CD8α^+^ cells population in the CALT of both the 63140- and CEO-inoculated birds were numerically increased at 1 dpi, decreased at 3 dpi, and increased again at 5 dpi, but were not significantly different from the mock-inoculated birds. For the 63140-inoculated birds, the CD8α^+^ cells significantly (*p* ≤ 0.05) decreased at 7 dpi, followed by a significant (*p* ≤ 0.05) increase at 9 dpi. For the CEO-inoculated birds, the CD8α^+^ cells percentages were sustained above mock levels from 5 to 9 dpi (Figure 5c) but were not significantly different to the mock-inoculated birds. In the HG, no significant differences in CD8α^+^ cells were detected from 1 to 9 dpi within CEO- and 63140-inoculated birds.

### 3.6. Overexpression of Granzyme A and Association with Viral Genome Load Decrease in CALT

**Figure 6:**
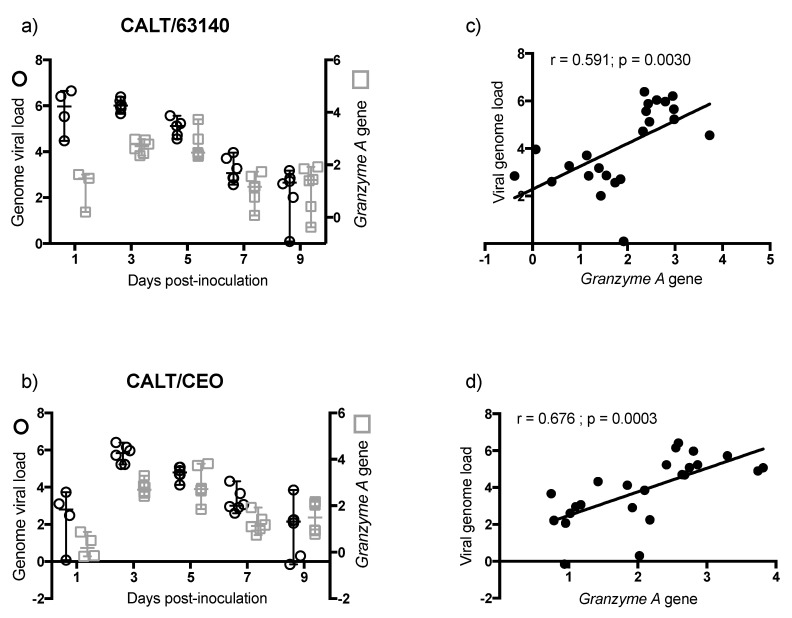
Transcription of the *Granzyme A* gene in relation to genome viral load in the conjunctiva-associated lymphoid tissue (CALT). Individual median fold change (log_10_ 2^−ΔΔ*C*t^) transcription of the *Granzyme A* gene in the CALT of 63140 (**a**) and CEO (**b**) groups are presented in the y right axis (open square), with the median represented as the horizontal line and the 95% confident interval as the vertical line. Individual mean genome load (log_10_ 2^−ΔΔ*C*t^) in the CALT of 63140 (**a**) and CEO (**b**) groups are presented in the y left axis (open circle), with the mean represented by the horizontal line and the standard deviation as the vertical line. Pearson correlation between *Granzyme A* expression and genome load from 3 to 9 dpi were determined for CALT/63140 (**c**) and CALT/CEO (**d**). Pearson correlation and linear regression analysis for x = *Granzyme A* gene expression and y = viral genome load was performed for 63140 (**c**) and CEO (**d**) groups. Correlation coefficient (r) and *p* values are presented.

The transcription level of the *Granzyme A* gene and viral genome load for 63140 and CEO groups in the CALT are shown in Figure 6. *Granzyme A* gene expression for both 63140 (Figure 6a) and CEO (Figure 6b) groups peaked between 3 and 5 dpi and the onset of virus genome decline started at 5 dpi. Changes in the *Granzyme A* gene expression from 3 to 9 dpi showed a significant correlation within the 63140 (r = 0.591, *p* = 0.003) (Figure 6c) and CEO (r = 0.6758, *p* = 0.0003) groups (Figure 6d).

### 3.7. Overexpression of Granzyme A and Association with Viral Genome Load Decrease in HG

**Figure 7:**
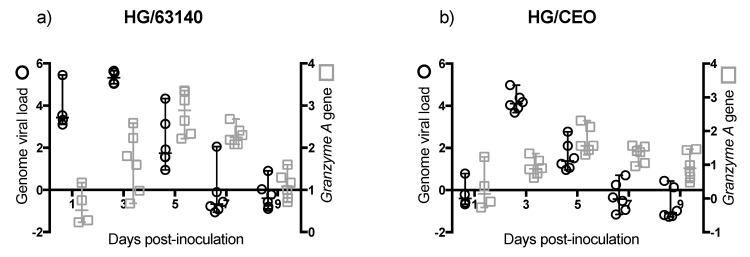
Transcription of the *Granzyme A* gene in relation to genome viral load in the Harderian gland (HG). Individual median fold change (log_10_ 2^−ΔΔ*C*t^) transcription of the *Granzyme A* gene in the HG of 63140 (**a**) and CEO (**b**) groups are presented in the *y* right axis (open square), with the median represented as the horizontal line and the 95% confident interval as the vertical line. Individual mean genome load (log_10_ 2^−ΔΔ*C*t^) in the CALT of 63140 (**a**) and CEO (**b**) groups are presented in the *y* left axis (open circle), with the mean represented by the horizontal line and the standard deviation as the vertical line.

The transcription level of the *Granzyme A* gene and viral genome load for 63140 and CEO groups in the HG are shown in Figure 7. *Granzyme A* gene expression peaked at 5 dpi, which coincided with the onset of 63140 and CEO genome load decline. However, by 7 dpi, the *Granzyme A* gene expression remained high, particularly for the 63140 group, while viral genome load declined in both the 63140 (Figure 7a) and CEO (Figure 7b) groups. No significant correlation was detected between *Granzyme A* gene expression and the decrease in 63140 or CEO viral genome load in the HG.

### 3.8. Variable Expression of IL-12p40 and IFN-γ Genes in the CALT and HG

**Figure 8:**
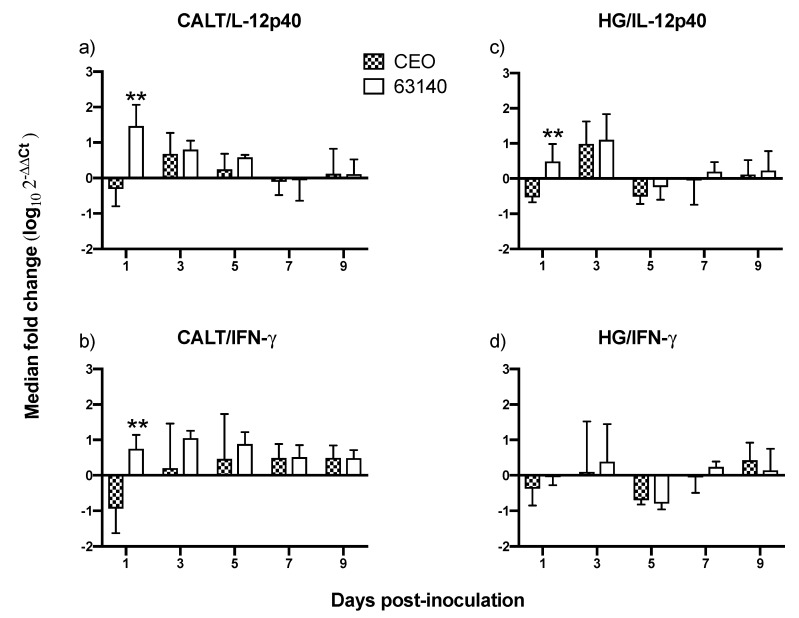
The transcription of *IL-12p40* and *IFN-γ* genes in conjunctiva-associated lymphoid tissue (CALT) and Harderian gland of chickens inoculated via the ocular route with CEO vaccine strain and virulent 63140 strain. The median fold change (log_10_ 2^−ΔΔ*C*t^) for *IL-12p40* transcription in the CALT (**a**) and in the HG (**c**), and transcription of the *IFN-γ* gene in the CALT (**b**) and HG (**d**) are presented as the median (bars) with 95% confident interval (lines). Differences in median fold changes for *IL-12p40* and *IFN-γ* between CEO and 63140 groups were determined by non-parametric Mann–Whitney U test (*p ≤* 0.05). Significant increase (*p ≤* 0.05) is indicated by (**).

*IL-12* is a pro-inflammatory cytokine which stimulates the induction of the *IFN-γ* protein. *IFN-γ* subsequently enhances phagocytic activity with the further release of IL-12 and other proinflammatory cytokines from mononuclear phagocytes. Therefore, a coordinated expression of *IL-12* and *IFN-γ* is required to elicit a strong, balanced cell-mediated immune response. Transcription of the *IL-12p40* gene in the CALT at 1 dpi for the 63140-inoculated chickens was significantly increased (*p* ≤ 0.05) compared to the CEO-inoculated birds. *IL-12p40* was downregulated in CEO-inoculated birds. The *IL-12p40* gene transcription in both the 63140- and CEO-inoculated birds increased on 3 and 5 dpi. Its expression decreased afterwards on 7 and 9 dpi (Figure 8a). Similarly, at 1 dpi, the transcription of the *IFN-γ* gene was significantly (*p* ≤ 0.05) higher for the 63140 birds than for the CEO birds. The 63140-inoculated birds had a peak of *IFN-γ* expression on day 3 pi, followed by a decrease from 7 to 9 dpi. The *IFN-γ* gene transcription for the CEO birds from 3 to 5 dpi was lower than that of the 63140 birds, but they were not significantly different. (Figure 8b). The most pronounced changes in *IL-12p40* and *IFN-γ* gene expression in the CALT occurred from 1 to 5 dpi for either group of infected birds. During this period, the expression of *IL-12p40* and *IFN-γ* for the 63140 birds showed no correlation. However, in the CEO birds, the expression of *IL-12p40* and *IFN- γ* genes yielded a strong correlation (r = 0.625, *p* = 0.0096).

In the HG, at 1 dpi, the *IL-12p40* gene transcription for the 63140-inoculated birds was significantly (*p* ≤ 0.05) increased compared to the CEO-inoculated birds. In the CEO-inoculated birds, *IL-12p40* was downregulated. For both the CEO- and 63140-inoculated birds, the *IL-12p40* gene transcription increased at 3 dpi and declined at 5 dpi (Figure 8c). Transcription of the *IFN-γ* gene in the HG was downregulated at 1 dpi for both CEO- and 63140-inoculated birds (Figure 8d). The clearest changes in *IL-12p40* and *IFN-γ* genes expression induced by these viral strains occurred from 1 to 5 dpi. During this period, the expression of both genes exhibited a strong correlation within the 63140 (r = 0.781, *p* = 0.0004) and the CEO (r = 0.581, *p* = 0.0182) groups.

## 4. Discussion and Conclusions

Although it is well documented that resistance against ILT is mediated primarily by cell-mediated immunity, little is known about the dynamics of immune cells in the mucosal tissues, or the factors that contribute to the development of host disease resistance. To begin to decipher the key immune components that function in mucosal tissues that contribute to disease resistance, we evaluated changes in (i) four subsets of lymphocyte populations (CD4, CD8 IgM^+^. And IgA^+^), (ii) transcriptional levels of *interleukin (IL)-12p40*, *interferon gamma (IFN)-γ* and *Granzyme A* genes in the eye-associated lymphoid tissue (CALT and HG) following ocular inoculation with the CEO vaccine or the virulent 63140 strain, and their correlation with viral genome load in the tissues.

The strong correlation between IgM^+^ B and MHCI^+^/MHCII^Hi+^ cells in the CALT for both the 63140- and CEO-inoculated chickens was indicative of an existing predominant subset of B cells expressing MHCI^+^/MHCII^Hi+^ in the tissues. It is possible that the increase in IgM^+^ and MHCI^+^/MHCII^Hi+^ B cells at 3 dpi reflects enhanced MHC class II-restricted antigen presentation, while the decline in this population at 7 and 9 dpi may indicate that activated B cells were undergoing isotype switching to IgY [26]. If this assumption is correct, the data would suggest that isotype switching occurred earlier for the 63140- (7 dpi) than for the CEO-inoculated birds (9 dpi). Even though IgY^+^ cells were not measured in this study, early (3 dpi) and late (9 dpi) increases in IgA^+^ B cells were detected. This suggests that an antibody response was mounted within the CALT of birds inoculated with CEO or 63140. Although the antigen specificity of the IgA^+^ B cells was not determined, it should be noted that the increase in IgA^+^ B cells occurred at the peak of (3 dpi) and following the decline (9 dpi) in virus replication in the conjunctiva epithelium. In earlier studies, it was determined that the peak of IgA^+^-producing cells in the trachea occurred at day 7 pi. This was after ILTV replication was no longer detected [27]. Also, it has been demonstrated that the immunomodulating effect of the viral chemokine binding protein, glycoprotein G, favors the recruitment of B cells over T cells to the trachea. This results in an enhanced humoral response, as indicated by an increase in circulating antibodies against ILTV [28]. Consequently, this immune modulation intensifies the level of the cytopathological lesions in the trachea epithelium [10]. The current study supports the premise that the immunomodulating effect exerted by the viral glycoprotein G favors ILTV persistence. Prolonged persistence of viral replication, and its associated damage to the conjunctiva epithelium that was observed following ocular exposure to virus. This was most clearly seen with the virulent strain, 63140. In the HG, the increase in the number of IgM^+^ and MHCI^+^/MHCII^Hi+^ cells also supported the activation of B cells. However, a concurrent decline in viral genome load in the HG corresponded with the decline in the number of IgA^+^ B cells in both groups of inoculated birds. This suggests that local IgA antibody response may not contribute significantly to the declined in virus replication. As the function of IgA is primarily to block the entry of the virus into cells at body surfaces, this was not completely unexpected. However, it has been reported that IgA^+^ B cells produced in the HG migrate to other tissue sites to influence the humoral immune response on other mucosal tissues [29].

The CD4^+^ T cells population in the CALT declined during the peak of virus replication. This decline was more severe for the 63140-inoculated birds than for the CEO-inoculated birds. In a previous study, after ocular inoculation with infectious bronchitis virus (IBV), a CD3^+^/CD44^+^ cells population, a phenotype characteristic of activated T cells in birds, declined in the CALT at 3 dpi, but reached its peak expansion by 11 dpi. The authors indicated that following IBV exposure effector T cells initially left the CALT, then during the immune expansion phase, the CD3^+^/CD44^+^ cells repopulated the CALT [16]. The CD4^+^ T cells population in the CALT of the 63140-inoculated birds expanded by day 7 pi. This coincided with the decline in viral genome load. However, viral lytic replication persisted in the conjunctiva epithelium during this time period. For the CEO-inoculated birds, the clearance of viral lytic replication from the conjunctiva epithelium occurred independent of a CD4^+^ cells expansion in the CALT. Taken together, these results suggest that CD4^+^ cells did not play a key role in the early clearance of the virus from the conjunctiva. In the HG, although no correlation between the number of CD4^+^ cells and decrease in viral genome load was revealed, the peak expansion of CD4^+^ cells (at 5 dpi) coincided with the decline in the genome viral load for each of the strains.

The transcription of the *Granzyme A* gene peaked at 3 dpi. This was prior to the expansion of the CD8α^+^ cells population. This was also during a significant contraction of CD4^+^ cells. This suggests that CD8α^+^ NK cells [30] may be activated at 3 dpi in the CALT. At 5 dpi, the enhanced expression of *Granzyme A* in the 63140 and CEO birds coincided with the onset of CD8α^+^ cell expansion. The CD8α^+^ cell population in the 63140 birds underwent a contraction at 7 dpi. This coincided with persisting lytic viral replication in the conjunctiva epithelium. In contrast, in the CEO birds, the CD8α^+^ population underwent a sustained, but gradual, expansion from 5 to 9 dpi. This coincided with the decline in viral genome load of CEO, and the decline in lytic viral replication in the conjunctiva. Independent of the number of CD8α^+^ cells in the tissue, changes of *Granzyme A* gene expression in the CALT strongly correlated with the decline of CEO and 63140 genomes.

Although no major change in the number of CD8α^+^ cells was detected in the HG, the enhanced expression of the *Granzyme A* gene indicated that CD8α^+^ cells were activated. However, in the HG, an increase in *Granzyme A* gene expression did not correlate with the decrease in viral genome load. In contrast to the epithelium associated with the CALT, the epithelium associated with the HG does not support lytic ILTV replication. However, viral antigen is found in association with mononuclear cells in the gland [17]. Whether the clearance of viral genomes in the HG at 7 dpi is the outcome of cytotoxic T cell activation, or to the migration of viral antigen positive mononuclear cells warrants further investigation.

An effective antigen-specific T cell response is contingent on a balanced and timely innate response. *IL-12* is a critical proinflammatory cytokine involved in the initiation and expansion of antigen-specific adaptive immune responses. *IL-12* acts in the pro-inflammatory cascade during inflammation. It is involved in the activation of both NK cells and cytotoxic T cells activity. IL-12 is a strong inducer of *IFN-γ* production. *IFN-γ* in turn enhances phagocytic activity of macrophages and monocytes and promotes the further release of *IL-12* and other proinflammatory cytokines. In mammals, bioactive *IL-12* is found in two splice forms, p40 and p35. The p40 form is primarily expressed by monocytes, macrophages and dendritic cells [31]. It is not currently known if chicken antigen presenting cells (APCs) produce *IL-12p40*. However, it has been confirmed that following stimulation with lipopolysaccharide (LPS), or unmethylated CpG DNA, chicken macrophages from peripheral blood, or bone marrow [32], and chicken thrombocytes [25] appear to express a functional analog of the *IL-12p40* gene.

In this study, the increased expression of the *IL-12p40* and *IFN-γ* genes at 1 dpi, and the increased expression of *IFN-γ* during the acute phase of the infection (3 to 5 dpi) in the CALT were not sufficient to neutralize the virus infection with the virulent 63140 strain. Whereas, in the CEO-inoculated chickens, the downregulation of *IL-12p40* and *IFN-γ* genes at 1 dpi appeared to elicit a more dampened inflammatory response. This was associated with the accelerated clearance of the virus lytic replication. In the HG, only the moderate expression of *IL-12p40* and *IFN-γ* at 1 and 3 dpi resulted in the expansions of both CD4^+^ and CD8α^+^ cell populations which appeared sufficient to limit viral replication in chickens inoculated with either virus.

As evidenced by the differential expression of *IL-12p40* and *IFN-γ* genes observed at the initiation of viral infection, it appears that local innate immune activation plays a significant role in governing the speed and efficacy of the adaptive immune response to ITLV. It has been demonstrated that highly pathogenic avian influenza H5N1 viruses [33] and velogenic Newcastle disease virus (NDV) [34] compromise the activation of NK cells. In addition to NK cells, another population of CD8α^+^ cells that are also capable of lysing virus infected cells independent of MHC restriction is a subpopulation of γδ T cells [35]. An approach to phenotyping and mapping the functional activities of all relevant CD8^+^ cells in CALT and HG will be required to identify the role that each group of CD8^+^ cells play in controlling lytic viral replication.

Finally, regulation of macrophage activity by Th1 and Th2 cytokines appears to be an important component in the balance of the immune response induced during ILTV infection. This balance is necessary to avoid the damage associated with excessive inflammatory activation. The injection of *IFN-γ* into mice prior to ocular inoculation with Herpesvirus simplex 1 (HSV-1) significantly increased virus replication in the eye, increased the number of latently infected trigeminal ganglia, and produced more severe lesions in the cornea [36]. Under this model, it was determined that macrophage polarization, provoked by *IFN-γ*, enhanced the production of inflammatory cytokines, and consequently exacerbated the disease. The macrophages in CALT and HG were not included in this analysis. Yet, the observed early increase in *IL-12p40* and *IFN-γ* genes expression induced by the 63140 strain in the CALT was followed by an increase in inflammatory infiltrates in the conjunctiva. These observations warrant an investigation of whether the virulent strain 63140, but not the CEO vaccine strain, favors the pro-inflammatory polarization of macrophages in the associated ocular tissues.

Taken together, this study attempted to document the dynamics of lymphoid cell populations in the two major ocular mucosal lymphoid organized tissues of the chicken during infection with a strongly virulent ILTV strain (63140) and a relatively attenuated vaccine virus, the CEO commercial vaccine virus. The study demonstrated that after ocular inoculation the severity and duration of the lesions in the conjunctiva epithelium induced by the CEO vaccine strain were limited and resolved faster than those induced by the 63140 virulent strain. The CALT actively responded to both viral strains. Each strain induced the generation of IgA^+^ cells and an increase in the number of IgM^+^ and MHCI^+^/MHCII^Hi+^ cells. This suggested that the B cells activation in the tissues examined was part of the adaptive immune response to ILTV. However, the immunomodulating factor in ILTV appears to promote this B cell activation bias. An increase in the transcription of *Granzyme A* and its association with the decline in the number of viral genomes in the tissue appears to suggest that cytotoxic T cell and NK cell activation, rather than activated B cells, was primarily responsible for the clearance of the virus infection from the conjunctiva. Virus clearance from the HG appeared to be mediated by a broader set of immune responses. These include the generation of IgA^+^ cells, the expansion of CD4^+^ cells, and the production of *Granzyme A* by resident CD8α^+^ cells. Rapidly after infection (1 dpi), the 63140 strain stimulated an upregulation of the *IL-12p40* and *IFN-γ* genes in the CALT, but the CEO vaccine strain downregulated the expression of both of these genes. Therefore, a surge in pro-inflammatory cytokine production, which sustains the antigen-specific cell-mediated response, did not accelerate the clearance of the virus from the conjunctiva. This outcome underscores the effect that interactions between innate cells and ILTV have on the robustness and speed of adaptive immune responses.

In conclusion, this work is the first to examine the changes in lymphoid cells within the eye-associated lymphoid tissue during the course of ILTV infections of different virulence. It supports the premise that ILTV-mediated immunomodulation promotes a B cell response over the more productive responses of T cells. Further, it provides evidence that expansions of CD8α^+^ cells, with the concomitant expression of the *Granzyme A* gene, are one key to halting ILTV cytolytic replication in the conjunctiva. Ultimately, this study revealed that the early upregulation of *IL-12p40* and *IFN-γ* cytokine genes was incompatible with the decline in virus replication during acute infection, thus enhanced development of lesions in the conjunctiva epithelium.

## Figures and Tables

**Table 1:** List of primers and the reaction conditions used for quantitation of host genes expression by real-time PCR.

			Real Time PCR Reaction					
Gene		Primers (5’-3’)	Den ^c^	Ann ^d^	Ext ^e^	Cycles	Eff ^f^	Reference
*β-actin*	Fw ^a^	CAACACAGTGCTGTCTGGTGGTA	95 °C (10 s) ^g^	60 °C (5 s)	72 °C (10 s)	45	2	[23]
	Rv ^b^	ATCGTACTCCTGCTTGCTGATCC						
*IFN-γ*	Fw	ACACTGACAAGTCAAAGCCGCACA	95 °C (10 s)	60 °C (5 s)	72 °C (10 s)	45	1.98	[23]
	Rv	GTCGTTCATCGGGAGCTTGGC						
*Granzyme*	Fw	TGGGTGTTAACAGCTGCTCATTGC	95 °C (10 s)	55 °C (5 s)	72 °C (10 s)	50	2.02	[24]
	Rv	CACCTGAATCCCCTCGACATGAGT						
*IL-12p40*	Fw	CCAAGACCTGGAGCACACCGAAG	95 °C (10 s)	60 °C (5 s)	72 °C (10 s)	50	2.0	[25]
	Rv	CGATCCCTGGCCTGCACAGAGA						

^a^ Forward primer; ^b^ Reverse primers; ^c^ Denature temperature; ^d^ Annealing temperature; ^e^ Extension temperature; ^f^ Efficiency, ^g^ Numbers in parentheses represent seconds.
